# Cyanidin 3-*O*-galactoside: A Natural Compound with Multiple Health Benefits

**DOI:** 10.3390/ijms22052261

**Published:** 2021-02-24

**Authors:** Zhongxin Liang, Hongrui Liang, Yizhan Guo, Dong Yang

**Affiliations:** 1 Beijing Key Laboratory of Functional Food from Plant Resources, College of Food Science & Nutritional Engineering, China Agricultural University, Beijing 100083, China; SY20193061085@cau.edu.cn (Z.L.); lhr_stem@cau.edu.cn (H.L.); guo_yizhancau@163.com (Y.G.); 2 Key Laboratory of Viticulture and Enology, Ministry of Agriculture and Rural Affairs, Beijing 100083, China; 3 Xinghua Industrial Research Centre for Food Science and Human Health, China Agricultural University, Xinghua 225700, China

**Keywords:** anthocyanin biosynthesis, galactosyltransferase, physiological functions

## Abstract

Cyanidin 3-*O*-galactoside (Cy3Gal) is one of the most widespread anthocyanins that positively impacts the health of animals and humans. Since it is available from a wide range of natural sources, such as fruits (apples and berries in particular), substantial studies were performed to investigate its biosynthesis, chemical stability, natural occurrences and content, extraction methods, physiological functions, as well as potential applications. In this review, we focus on presenting the previous studies on the abovementioned aspects of Cy3Gal. As a conclusion, Cy3Gal shares a common biosynthesis pathway and analogous stability with other anthocyanins. Galactosyltransferase utilizing uridine diphosphate galactose (UDP-galactose) and cyanidin as substrates is unique for Cy3Gal biosynthesis. Extraction employing different methods reveals chokeberry as the most practical natural source for mass-production of this compound. The antioxidant properties and other health effects, including anti-inflammatory, anticancer, antidiabetic, anti-toxicity, cardiovascular, and nervous protective capacities, are highlighted in purified Cy3Gal and in its combination with other polyphenols. These unique properties of Cy3Gal are discussed and compared with other anthocyanins with related structure for an in-depth evaluation of its potential value as food additives or health supplement. Emphasis is laid on the description of its physiological functions confirmed via various approaches.

## 1. Introduction

**Figure 1:**
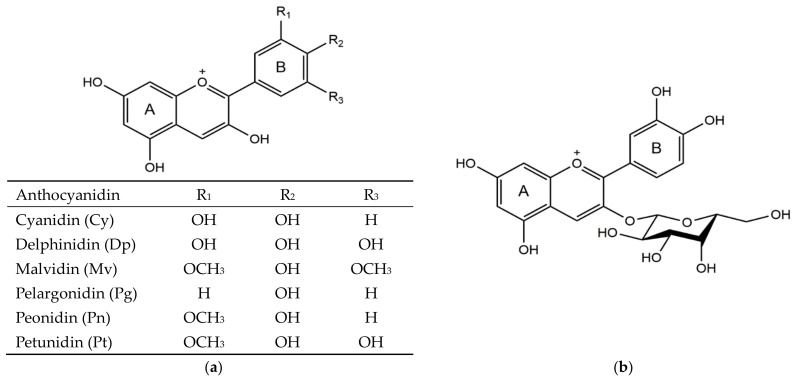
Structures of six common anthocyanidins (**a**) and cyanidin 3-*O*-galactoside (**b**).

Anthocyanins are water-soluble chemicals consisting of an aglycone (anthocyanidin), sugar(s), and, in many cases, acyl group(s) [1]. They are categorized as parts of flavonoids that have similar structures. Most identified anthocyanins are based on the six most common anthocyanidins, as shown in Figure 1a. Anthocyanins naturally present in a wide range of plant tissues, including flowers, fruits, leaves, roots, stems, and so forth. They work as pigments, which provide cyanic colors for these parts of the plants [1]. Anthocyanins are also critically important to plant physiology; for instance, anthocyanins often produce protective effects for plants against extreme temperature conditions, and regulate the light absorption of photosynthetically active tissues, which further affect the metabolism of the whole plant [2,3,4]. Given that many parts of a common daily diet are good sources of anthocyanins, an enormous number of studies were performed to investigate their physiological activity in model animals as well as in humans during recent decades [5]. It has been well recognized that the consumption of anthocyanin is highly related to the reduced risk of diseases, such as cancer, atherosclerosis, cardiovascular diseases, and diabetes [6,7,8,9].

Cyanidin 3-*O*-galactoside (Cy3Gal) (Figure 1b) belongs to the group of glycosides of cyanidin, which is the most widespread anthocyanidin in the plant kingdom [10]. Cy3Gal mainly occurs in fruits, including red-skinned or red-fleshed apples, hawthorn, bilberries, cranberries, chokeberries, and lingonberries [11,12,13,14,15,16,17]. As these fruits have the potential to be an important part of the human diet (in the form of juice, for example), research has been conducted to explore the biosynthesis, chemical stability, natural occurrences, extracting methods, physiological functions, and prospective applications of Cy3Gal. Particularly, in recent years, Cy3Gal was heighted for its excellent ability to work as a cognitive improver [18,19]. However, there is no report comprehensively describing all of the scientific discoveries about this compound. Based on previous studies, here we collectively conclude the information related to these aspects of Cy3Gal. Therein the description of its physiological functions is given in details for a better evaluation of its value as a nutrient.

## 2. Biosynthesis

**Figure 2:**
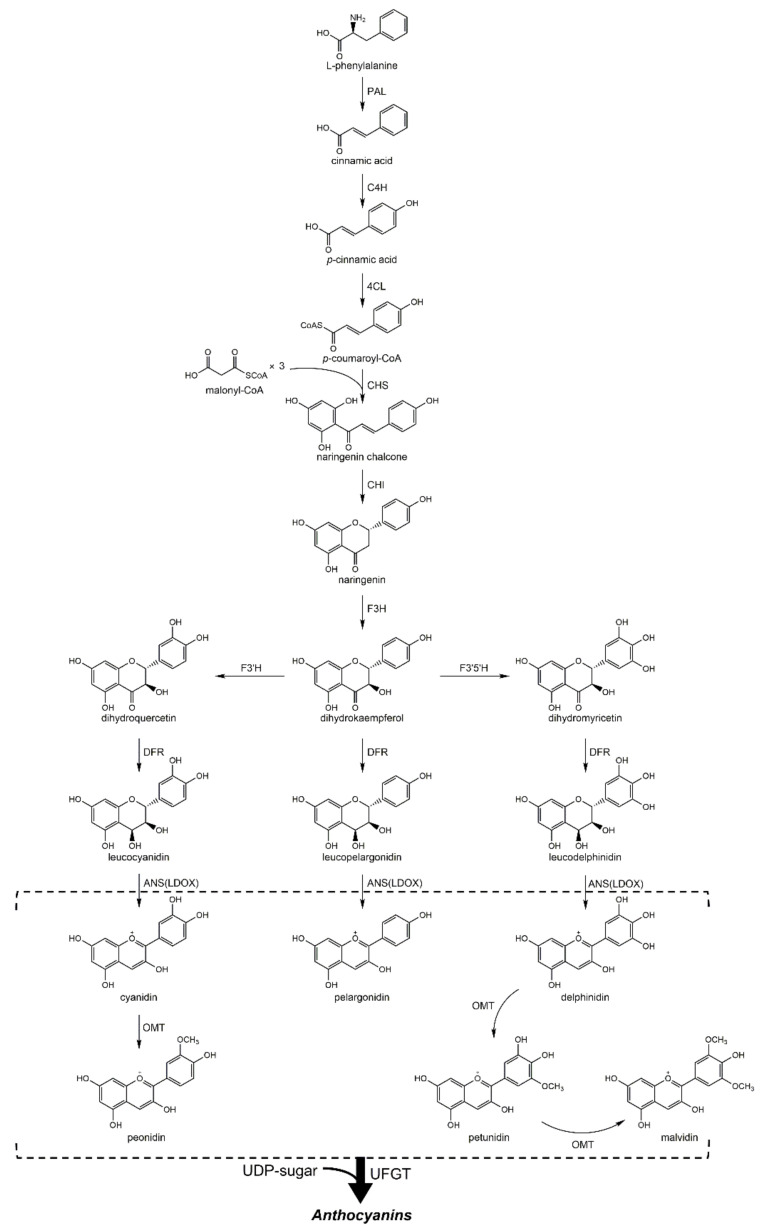
Biosynthesis pathway of anthocyanins.

**Figure 3:**
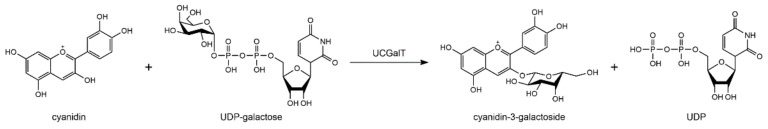
Formation of cyanidin 3-*O*-galactoside from cyanidin and UDP-galactose.

According to previous studies, the biosynthesis of Cy3Gal and other anthocyanins in plants follows a uniform pathway, in which phenylalanine act as the initial compound for a series of following chemical reactions (Figure 2) [20,21]. Phenylalanine ammonia lyase (PAL) is the first enzyme participating in the anthocyanin biosynthesis pathway. In the presence of PAL, phenylalanine catalytically transforms into *trans*-cinnamic acid via *trans*-elimination of ammonia. Then the cinnamic acid successively changes to *p*-coumaric acid and *p*-coumaroyl-CoA as mediated by cinnamate 4-hydroxylase (C4H) and 4-coumarate-CoA ligase (4CL), respectively. The pathway continues with the chalcone synthase (CHS)-catalyzed formation of naringenin chalcone from three molecules of malonyl-CoA and one molecule of *p*-coumaroyl-CoA. Subsequently, the stereo specific and rapid isomerization of naringenin chalcone into naringenin is catalyzed by chalcone isomerase (CHI). The following steps result in the synthesis of three dihydroflavonols: dihydrokaempferol (DHK), dihydroquercetin (DHQ), and dihydromyricetin (DHM). Specifically, flavanone 3-hydroxylase (F3H) reacts with naringenin and converts it into DHK, which then can be either hydroxylated at the 3′ position (by F3′H) or at both the 3′ and 5′ positions (by F3′5′H) to produce DHQ and DHM, respectively. Following, the three dihydroflavonols are switched by dihydroflavonol reductase (DFR) by stereospecific reduction to, separately, three different colorless leucoanthocyanidins: leucopelargonidin (from DHK), leucocyanidin (from DHQ), and leucodelphinidin (from DHM). Subsequent reactions in the participation of anthocyanidin synthase (ANS), also known as leucoanthocyanidin dioxygenase (LDOX), convert these leucoanthocyanidins to the colored anthocyanidins (pelargonidin, cyanidin, and delphinidin). These happen before their combinations with sugars in the uridine diphosphate (UDP)-binding form with the help of UDP-sugar:flavonoid 3-*O*-glycosyltransferase (UFGT). In particular, in the presence of UDP-galactose, UDP-galactose: cyanidin galactosyltransferase (UCGalT) can induce the production of Cy3Gal from cyanidin via glycosyl transformation (Figure 3) [22,23,24,25].

UDP-galactose is mainly from the intermediates in the production of polysaccharides via nucleotide sugars metabolism in plants [26]. It can be synthesized through two routes: from UDP-glucose mediated either by UDP-glucose 4-epimerase or by galactokinase and galactose-1-phosphate uridyl transferase in the presence of galactose [27]. Additionally, *O*-methyltransferase (OMT) catalyzes the formation of malvidin, peonidin, petunidin, and their corresponding glycosylated forms from cyanidin, delphinidin, and corresponding anthocyanins (Figure 2) [28]. After their biosynthesis, these flavonoids would be transported to the vacuoles or cell walls of plants [29].

The regulation of the anthocyanin biosynthesis at the cellular level is mainly attributed to two classes of genes that were identified in many species. One class is the structural gene encoding enzymes, which directly participate in the catalysis of each step in the pathway; the other class is those encoding the transcription factors that mediate the expression of these structural genes [30]. Different states of these structural genes (silencing, normal expression, over expression, or heterologous expression) of the involved enzymes impact the final flavonoid composition in plant tissues [31,32,33,34,35,36]. Hence, the actions of the transcription factor genes are important to the correct anthocyanin accumulation during plant development. To date, three groups of transcription factors, namely MYB, bHLH, and WD40 proteins, were identified, and they are respectively homologous among different plant species [37]. These transcription factors often work in the form of complexes, and are affected by both the developmental regulation factors (e.g., plant hormones, such as abscisic acid and ethylene) and the environmental control factors (e.g., light intensity and temperature) [28]. However, this regulation mechanism is general for the biosynthesis of all anthocyanins. Only the genes encoding the enzyme UCGalT are specific to the biosynthesis of Cy3Gal, and they were characterized in plants such as *Malus domestica*, *Apium graveolens*, *Daucus carota*, *Vigna mungo*, *Petunia hybrida*, and *Actinidia chinensis* (Figure S1) [22,24,25,38,39,40]. An upregulated expression of these genes could directly result in the increased amount of Cy3Gal in the plants.

## 3. Chemical Stability

Anthocyanidins are naturally unstable, and their stability is greatly affected by environmental factors, such as pH and temperature [41]. Glycosyl addition improves the stability of anthocyanidins via diverse mechanisms, such as forming H-bonds, van der Waals interactions, enhancing steric hindrance, and decreasing electron delocalization. Thus, most of the anthocyanidins are found glycosylated (anthocyanins) in nature [42]. The reactivity and stability of anthocyanins vary according to their chemical composition, which is the anthocyanidin, glycoside, and each of their substitution sites on the anthocyanidin molecule [43].

Cy3Gal is a typical mono-glycosylated anthocyanin susceptible to heating in food processing and rising temperature during food storage. Red-fleshed fruits of *Malus* profusion stored at 33 ± 2% for 1 week led to a decrease of more than 50% in Cy3Gal content [44]. Storage at 70% for 24 h led to a roughly 50% decrease in the total anthocyanin contents of freeze-dried aronia fruit (chokeberry) powder in which Cy3Gal is the predominant anthocyanin (about 65%) [45,46,47]. Cryogenic storage prolongs the Cy3Gal preservation time of food that contains this compound, but this also relies on other environmental factors. For instance, early study of Lin et al. [48]. More than 90% of the total Cy3Gal in *Starkrimson* apples remained after a 30-week storage at 2% and 73% relative humidity. This is consistent with the work of Zhang et al. on *Fuji* apple peels, which also demonstrated the positive effects of high humidity on the preservation of Cy3Gal [49]. In aqueous solution, acidity is another major impact factor. Although anthocyanins are generally more stable in acidic solutions than in alkaline solutions, approximately 40% of Cy3Gal in aronia juice (pH = 3.5) would be lost after 2-month storage at 4% [50,51]. Similarly, an unsatisfying stability of Cy3Gal in acidic solution (red-colored apple juice, pH < 3.0) was also reported by Knebel et al. [52]. Besides acidity, light also accelerates the degradation of synthesized anthocyanins [41]. Cy3Gal is naturally photosensitive—red-fleshed apple anthocyanins, of which ~75% is Cy3Gal, yielded a half-life of 6.42 days under natural daylight in aqueous solution at 30% [53]. Around 60% of Cy3Gal in solution degraded after ultraviolet (254 nm) irradiation for 20 min [54]. Mental ion is deemed as anthocyanin-stabilizing factor via mental complexation [41]. The work of Ngo and Zhao found that the Sn ion was one of the key reagents to stabilize red anthocyanins on pear peels (see the original for details), where Cy3Gal accounts for the largest part of total anthocyanins (above 60%) [55,56]. Moreover, oxygen content exhibits impact on the capability of the system to retain Cy3Gal. According to Awad and de Jager, the ultra-low oxygen condition storage at 1% for “Jonagold” (1.2% O_2_ + 5.0% CO_2_, for up to 8 months) and “Elstar” (1.2% O_2_ + 3.0% CO_2_, for up to 6 months) apples could maintain the concentration of Cy3Gal at a constant level during the storage, plus 1 or 2 weeks of shelf life [57]. On the other hand, the work of Zheng et al. showed that the blueberry fruit during the first 2–3 weeks of storage at 5%, oxygen level from 60 to 100%, provides a higher increasing effect (estimated at 3–4 times compared with the control after 2-week storage), and a lower decay rate (estimated at 15–50% of the decay rate of the control after 5-week storage) on the content of Cy3Gal, as well as other anthocyanins [58].

Compared to other anthocyanins, Cy3Gal exhibits difference in its chemical stability. For instance, in cranberry, the Cy3Gal was found to be more stable against oxygen and ascorbic acid degradation during storage than other anthocyanins with arabinose [59]. However, under heating conditions, Cy3Gal was proven to be less stable than cyanidin 3-arabinoside (Cy3Ara) [60]. Another previous study found that in blueberry, Cy3Gal was more stable against trifluoroacetic acid (TFA) mediated hydrolysis than Cy3Ara, but less stable when compared to cyanidin 3-glucoside (Cy3Glu) [61]. Meanwhile, at low acid concentration (≤1% TFA), Cy3Gal was the most stable as compared to malvidin 3-galactoside (Mv3Gal), petunidin 3-galactoside (Pt3Gal), and delphinidin 3-galactoside (Dp3Gal); however, at higher acid concentration (≥3% TFA), Cy3Gal was found to be the most unstable among these anthocyanins [61]. More recently, Farr et al. reported that Cy3Gal exhibited lower resistance to hydration and bleaching than Cy3Glu in solution under pH 1-pH 9 [42]. The analogous difference between the stability of these two compounds was also reported in alkaline solution by Sigurdson et al. [62]. These studies highlighted the differences between the chemical stability of Cy3Gal and that of other anthocyanins.

Many other external treatments upon food containing Cy3Gal had been proved to be capable of affecting the stability of this compound, such as adding exogenous additives, such as vitamin C and phenolic acids, high hydrostatic pressure treatment, vacuum-microwave and freeze drying, heat and microwave pretreatment, etc. [51,53,63,64,65]. Some of these methods have already been industrially employed.

## 4. Natural Occurrences and Extracting Methods

As stated previously, Cy3Gal resides in a wide range of natural sources, mainly in apples and berries. Numerous studies have been performed to evaluate the feasibility of different extracting methods against different plants. In this section, extracting methods of these studies are summarized in detail, and the natural sources of Cy3Gal used in these studies are concluded in Table 1.

According to previous studies, the extracting methods of Cy3Gal, coupled with other anthocyanins, are substantially similar: generally, the raw plant materials would be chopped or peeled into small pieces, depending on the distribution of anthocyanins, before being ground into fine powder using liquid nitrogen [44,66,67]. The powder was subsequently immersed in acidified organic solvent, normally methanol with hydrochloric acid or formic acid, to extract the anthocyanins inside the plant tissues. Shaking and sonicating were always employed in this step to improve the extraction yield. The mixture was then centrifuged to separate the supernatant and the residue. After filtration, the supernatant was analyzed with (ultra) high performance liquid chromatography (HPLC) and mass spectrum (MS) for quantitative and qualitative detection of anthocyanins. Alternatively, juices produced from fruits were also directly applied to the LC and MS analysis [12,52,67]. The commonly-applied analysis systems were often equipped with a C18 column in LC with organic solvents (e.g., formic acid, acetonitrile) as mobile phase for effective separation and a triple quadruple tandem MS with electrospray ionization (ESI) for accurate identification. Moreover, most of the studies mentioned in this paper were performed similarly as the above methods, with minor differences.

Cy3Gal is available from various plants (Table 1). As illustrated, the content of Cy3Gal varies with the species where it comes from. Berries, especially chokeberries, bilberries, and crowberries (Table 1) exhibit an outstanding capacity of holding high content of this compound. This indicates an ideal source for mass-extraction of Cy3Gal. Comparatively, Cy3Glu is mainly found in elderberries, blackberries, and Cy3Ara is also in chokeberries [68,69,70]. However, it should be noted that, environmental conditions under which plants grow have significant impacts on Cy3Gal accumulation during plant development. Such conditions inclusively interlink the effects from temperature, light exposure, air humidity, nutrition supplement, etc. [49,56,71,72,73]. What is more, minor differences during the extracting process could also lead to changes to the extraction yield of Cy3Gal from different sources. The information provided here is for reference purposes only.

## 5. Physiological Functions

**Figure 4:**
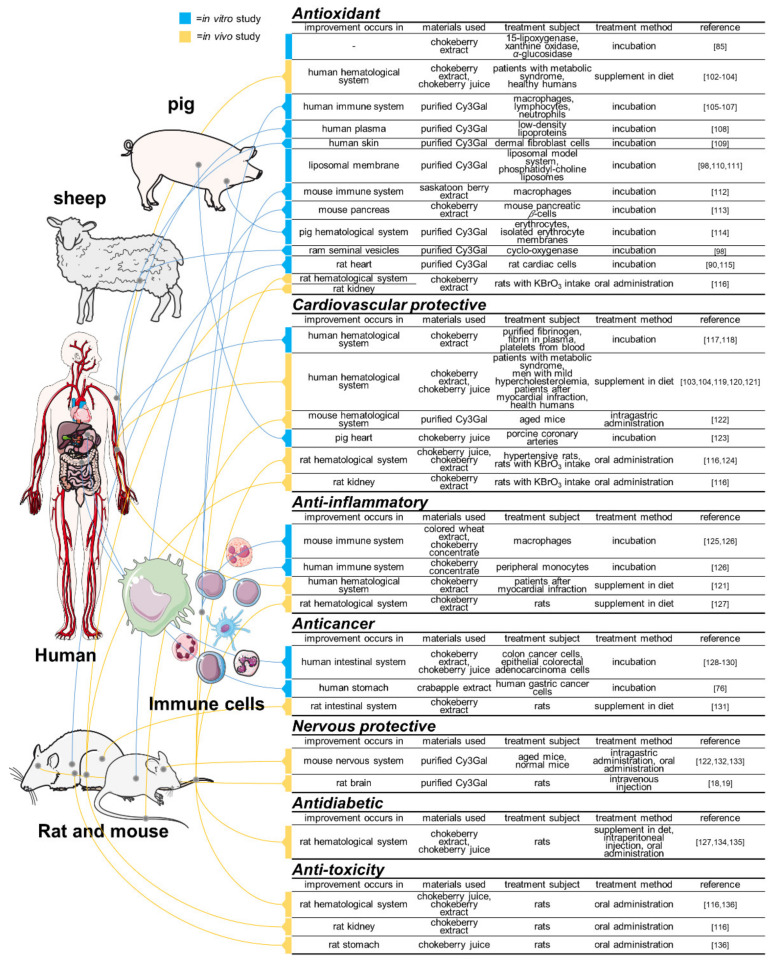
Studies in the physiological functions of cyanidin 3-*O*-galactoside and its enriched plant products. Blue color indicates the in vitro studies; orange indicates the in vivo studies. Precise induced physiological changes in the studied subjects of each studies are discussed in the text.

In this session, the physiological functions of Cy3Gal are introduced separately in two parts, sorted according to different experimental systems (in vitro or in vivo). According to the experimental materials used (purified Cy3Gal or products of plant) in the studies, each of the two parts is further separated into two aspects—acting solely and acting synergistically. This is used to differentiate that Cy3Gal performs the physiological functions with or without the presence of other compounds. In addition, the studies mentioned below are also reflected in Figure 4, categorized according to the specific health functions [18,76,85,90,98,102,103,104,105,106,107,108,109,110,111,112,113,114,115,116,117,118,119,120,121,122,123,124,125,126,127,128,129,130,131,132,133,134,135,136].

It should be noted that, compared with purified Cy3Gal, plant fruits or their extracts, juices, and concentrates are more widely employed in the in vivo studies. Specially, chokeberry (*Aronia melanocarpa*) has long been attracting much attention from researchers since it was valued as an important source of antioxidants, such as polyphenols, including phenolic acids and flavonoids. As this plant is particularly enriched in Cy3Gal, it is worth mentioning the studies related to chokeberry intake or treatment [47]. Other plants of interest are also included. It is worthy to note that studies are only selectively documented here for discussion of the potential role and synergistic health effects of Cy3Gal.

### 5.1. In Vitro Studies

#### 5.1.1. Acting Solely

Compounds that can donate free electrons or hydrogen atoms to reactive free radicals are always ideal antioxidants. Anthocyanin has long been deemed as the key factor contributing antioxidant capacity in many fruits and vegetables [137]. Cy3Gal as an individual bioactive substance has also been proven to hold superior capacities in DPPH (1,1-diphenyl-2-picrylhydrazyl) radical scavenging, ABTS (3-ethylbenzthiazoline-6-sulfonicacid) radical scavenging, hydrogen peroxide scavenging, ferric ion reducing, oxygen radical absorbance, and singlet oxygen quenching catalyzing [106,108,138,139]. In particular, previous work of Yan et al. highlighted the capacity of Cy3Gal extracted from cranberry fruit (*Vaccinium macrocarpon*) to reduce the cupric ion induced oxidation of low-density lipoproteins (low-density lipoprotein (LDL) + very-low-density lipoprotein (VLDL)) from plasma, measured with thiobarbituric acid reactive substances (TBARS, the indicators of lipid peroxidation) [108]. Adhikari et al. found that Cy3Gal purified from Saskatoon berries (*Amelanchier canadensis*) could impede the activity of cyclo-oxygenase (from ram seminal vesicles) and lipid peroxidation initiated by ferrous (Fe(II)) ion in a liposomal model system [98]. Such results are in accordance with the work conducted by Gabrielska and Oszmiański, who showed that Cy3Gal purified from chokeberries is capable of inhibiting lipid peroxidation induced either by Fe(II) ions, UV irradiation, or 2,2-azobis (2-amidinopropane) dihydrochloride (AAPH) peroxyl radicals, in a liposomal membrane system [110]. The above results were confirmed by the work of Adhikari et al. by monitoring the relative fluorescence intensity of the fluorescence probe, 3-(p-(6-phenyl)-1,3,5-hexatrienyl) phenylpropionic acid (DPH-PA), in the tested liposome membranes [98].

Study of Lyu and Park demonstrated the significant inhibition effect of Cy3Gal isolated from the fruits of *Acanthopanax divaricatus* Var. *albeofructus* on the negative oxidant effects induced by ultraviolet A (UVA) irradiation, such as H_2_O_2_ generation, lipid peroxidation, and antioxidant enzymes (e.g., superoxide dismutase (SOD) and catalase (CAT)) inactivation, on human dermal fibroblast cells (HDF-N) [109]. This is in accordance with the study of Lyu et al. conducted on H_2_O_2_-pretreated human macrophages (U937) utilizing Cy3Gal isolated from *Ogapy* (*Acanthopanax divaricatus* var. *albeofructus*) and similar testing methods (monitoring lipid peroxidation, activity of SOD and CAT) [105]. Similarly, Strugała et al. also affirmed that Cy3Gal could promote the protection of lipid membranes against the onslaught of free radicals, via experiments on phosphatidylcholine liposomes exposed to oxidation caused by AAPH [111].

Studies of Bellocco et al. revealed the protective effects of Cy3Gal against the tert-butyl hydroperoxide (t-BOOH)-induced peroxide damage in human lymphocytes [106]. They observed in the presence of Cy3Gal, lymphocytes incubated with t-BOOH exhibited a lower rate of lactate dehydrogenase (LDH) release (cell necrosis) and caspase 3 activation (cell apoptosis) compared with the control. Such results are consistent with the similar studies on rat cardiac cells (H9c2) exposed to oxidation-related ischemia-reperfusion injury, and H_2_O_2_-induced oxidative stress, respectively [90,115]. Moreover, studies of Bellocco et al. also found that Cy3Gal could alleviate the lymphocyte DNA damage from t-BOOH [106].

Recently, Denev et al. observed mild inhibitory effects of Cy3Gal (isolated from black chokeberries) on the production of reactive oxygen species (ROS) in human neutrophils when activated by phorbol myristate-acetate (PMA) [107]. However, such effects of Cy3Gal were not found, at least significantly, in neither spontaneous nor opsonized zymosan particles (OZP)-activated ROS production.

The binding constants of Cy3Gal for human cannabinoid receptors were determined by Korte et al. for an explanation of its antioxidative effects [140]. Bonarska-Kujawa et al. performed a series assays on pig erythrocytes, as well as isolated erythrocyte membranes, obtained from fresh blood to investigate the mechanism of the antioxidant action of Cy3Gal in respect of biological membranes [114]. It was observed with scanning electron microscope that Cy3Gal caused an inclination of erythrocytes to form echinocytes. The exact location of the postulated interaction between the studied compounds and erythrocyte membranes was examined by applying various membrane models, fluorescent probes 1,6-diphenyl-1,3,5-hexatriene (DPH), and 1-(4-trimethylammoniumphenyl)-6-phenyl-1,3,5-hexatriene *p*-toluenesulfonate (TMA-DPH), as well as Laurdan and Prodan probes. The selected fluorescent probes could incorporate at different depths of the hydrophobic region of the membrane, while Laurdan and Prodan probes are sensitive to polarity of the different phase states of lipid bilayers. It turned out that the observed protective actions of Cy3Gal on lipid membrane against ultraviolet C (UVC) irradiation- and AAPH-induced oxidation were based on its incorporation into the hydrophilic part of the membrane. It also stated that such incorporation probably formed a protective shield of the cell against external substances like free radicals [114].

Comparatively, Cy3Gal has its unique in vitro chemical, especially antioxidative properties. For example, the DPPH-radical scavenging activity of Cy3Gal was found to be the highest among the tested flavonoids including myricetin 3-galactoside, quercetin 3-galactoside, etc. [108]. The antioxidative activity of Cy3Gal tested by FRAP assay was higher than that of Pg3Glu, Mv3Glu, Pt3Glu, Pn3Glu, and Dp3Glu, but slightly lower than that of Cy3Glu [138]. On the other hand, in the work of de Rosso et al., the singlet molecular oxygen quenching ability of Cy3Gal was shown to be lower than that of Mv and its glycosides, but was less different with that of Cy3Glu [139]. In general, a compound’s antioxidative capacity is mainly related to its polarity, size, free -OH number, etc. It was believed that, for the antioxidative activity of monoglucosides, there was no significant difference observed between glucoside and galactoside, but arabinoside was found to be a weaker antioxidant [43]. However, such minor difference between Cy3Gal and Cy3Glu was not always duplicable in other studies, apart from simple antioxidative assays. For example, the activity of Cy3Gal to reduce t-BOOH-induced peroxide damage in human lymphocytes was much more significant than that of other anthocyanins including Cy3Glu [106]. Moreover, in the study of Isaak et al., for the oxidation-related ischemia-reperfusion injury on rat cardiac cells, the protective effects exerted by Cy3Glu were much higher than that by Cy3Gal, but less higher than that by Cy3Ara [90]. These indicate that the bioactivity of Cy3Gal is not in all respects similar with that of Cy3Glu.

#### 5.1.2. Acting Synergistically

Plants containing high amount of Cy3Gal were reported to yield strong antioxidant ability, though this is not only due to the presence of Cy3Gal. In the study of Wangensteen et al., extracts from four chokeberry cultivars (*Aronia melanocarpa* “Moskva”, “Hugin”, “Nero”, and *Aronia prunifolia*) exhibited high capacity of inhibiting peroxidative enzyme 15-lipoxygenase (15-LO), prooxidative enzyme xanthine oxidase (XO) and α-glucosidase in vitro [85]. The investigation by Rugină et al. into the antioxidant capacities of chokeberry (Nero) anthocyanin extracts demonstrated that the extracts could protect the mouse pancreatic *β*-cells (*β*TC3) against hydrogen peroxide- (H_2_O_2_-) and high glucose- (HG-) induced oxidative stress conditions [113]. The mechanism was explained as the induced improvement in antioxidant enzyme system including SOD, CAT, and peroxidase (GPx) [113].

The protective effects of polyphenols against cardiovascular diseases have also been the focal points of many studies. In the work by Auger et al., chokeberry juice was used to treat the isolated porcine coronary arteries, and was then identified as potent inducer of endothelium-dependent relaxations in the coronary arteries via monitoring the changes of its tension [123]. Such results suggest the vasorelaxant action of chokeberry products. The underlying mechanism of this action of chokeberry juice was discussed in their further work [141]. Malinowska et al. investigated the role of chokeberry extracts in the clot formation and the fibrinolysis in the model of hyperhomocysteinemia (induced by homocysteine) [117]. Results showed that chokeberry extracts decreased thrombin-induced fibrinogen polymerization in vitro and fibrin polymerization in human plasma system with and without the presence of homocysteine (Hcys) or the reactive form of the former, homocysteine thiolactone (HTL). Although chokeberry extracts did not cause changes in the tissue plasminogen activator (t-PA) catalyzed fibrin lysis in whole human plasma, it could otherwise lessen the fibrin lysis decrease caused by Hcys or HTL. Such results indicated the anticoagulant properties of chokeberry extracts. Furthermore, the antiplatelet properties of chokeberry extract were highlighted in the study of Olas et al., which illustrated that after the incubation of platelets isolated from human blood with chokeberry extract, significant decreases were manifested in three points: platelet adhesion to collagen, collagen–induced platelet aggregation, and the production of superoxide anion radicals [118].

Trumbeckaitė et al. illustrated that bilberry (*Vaccinium myrtillus*) fruit extracts enriched in Cy3Gal directly regulated the rat heart mitochondrial respiration rate in vitro [142]. However, the concomitant experiment in this study found no relation between purified Cy3Gal and the mitochondrial respiration with pyruvate plus malate as respiratory substrates.

The anti-inflammatory capability is deemed as an important part of anthocyanin physiological functions. Early study of Hu et al. proved that Saskatoon berry (*Amelanchier alnifolia* Nutt.) extracts impeded peroxyl radical-induced intracellular oxidation in mouse macrophage RAW264.7 cells [112]. Dark blue grained wheat (*Triticum aestivum* L. cv. Hedong Wumai) extracts rich in both Cy3Gal and Cy3Glu were proved to reduce the nitric oxide (NO) production in bacterial lipopolysaccharide (LPS)-activated RAW264.7 cells in the work of Hu, Cai, Li, Corke, and Kitts [125]. Likewise, Appel et al. observed that chokeberry concentrate inhibited both the release of proinflammatory cytokines (tumor necrosis factor α (TNF-α), interleukin 6 (IL-6), and IL-8) and the activation of inducible transcription factors (nuclear factor-κB that regulates the gene expression of pro-inflammatory cytokines) in LPS-incubated human peripheral monocytes and mouse macrophage RAW267.4 cells, respectively [126]. This indicated the anti-inflammatory capacity of plant extract or concentrate containing high amount of Cy3Gal. In the study by Martin et al., chokeberry extract also exhibited anti-inflammatory capacity on murine splenocytes by inhibiting LPS-stimulated IL-6 production [143]. However, further work using purified flavonoids suggested that the IL-6 inhibition observed with chokeberry extract may not be due to Cy3Gal, but the minor polyphenols, novel constituents, or synergies among these components. Additionally, according to the study of Xu and Mojsoska, the immunomodulation properties of chokeberry extracts in LPS-stimulated human monocytes mono mac 6 were highlighted according to the observed decreases in the TNF-α and IL-10 production, and in contrast to the studies mentioned above, an increase in the IL-6 production [144]. These were also deemed as attributing little to the anthocyanins contained in chokeberry by comparatively assessing the effects of anthocyanin-rich fraction and chokeberry crude extracts [144].

The anticancer ability of products from Cy3Gal enriched plants was also proven in vitro. Based on the studies of Malik et al. and Zhao et al., extract from chokeberry was shown to inhibit the growth as well as to stimulate apoptosis of human HT-29 colon cancer cells but exerted only minor effect on the growth of non-transformed NCM460 colonic cells [128,129]. Another study also indicated the ability of chokeberry juice to inhibit caco-2 cell proliferation by causing G2/M cell cycle arrest [130]. The phenolic extracts of crabapples (*Malus* “Royalty”), with Cy3Gal as predominance by more than 55% of total phenol, exhibited the ability to inhibit human gastric cancer cell (BGC-803) growth and induce their apoptosis [76]. It was believed that this was attributed to the phenol-induced changes in the expression of apoptosis-related genes, i.e., increase in *Bax* and *Bak* expression and decrease in *Bcl-2* and *Bcl-xl* expression [76]. However, Cy3Gal solely cannot inhibit human tumor cell growth as proven in the previous study of Zhang et al. [145].

### 5.2. In Vivo Studies

It should be noted that, it remains unclear whether all of the positive physiological effects that Cy3Gal possesses are still present or significant after human or animal digestion. In general, when biomolecule is released from food after human intake, it travels through the gastrointestinal tract and then gets absorbed into the bloodstream. Through the bloodstream, it can be further transported to target tissues where it provides health benefits. For instance, anthocyanins travel via the portal vein into the liver and then are distributed to hepatocytes. After finishing metabolism in the liver, anthocyanins may return to the intestinal system or move into general circulation before being removed by kidneys and excreted through urine [146]. However, anthocyanins are the biomolecules known with low bioavailability via the absorptive surface of the gastrointestinal tract, estimated in ranges of ~2%, with only trace quantities detected in the bloodstream or the target organs [146]. For example, Cy3Gal became detectable, with a highest detected rate as 0.45 ± 0.13 μg/mL in human blood serum 3 h after blueberries ingestion [147]. Moreover, in experimental animals, the concentrations of Cy3Gal in rat liver and kidneys 15 min after fed with bilberry extract are 2.43 ng/g tissue and 6.38 ng/g tissue, respectively [148]. Furthermore, it was also found that Cy3Gal presented in swine brain after blueberry ingestion [149,150]. After 3-week bilberry extract treatment, the detected content of Cy3Gal in different regions of swine brain ranged from 0 to 0.90 ng/g tissue [150]. It should be noted that the systemic metabolism of anthocyanins may cause bioavailability to be underestimated if bioassays only target parent compounds and phenolic acid breakdown products [151]. If taking into account the unmetabolized parent compound and all of the classes of metabolites, the total bioavailability of anthocyanins will be much greater than previously thought. However, details about how anthocyanins are absorbed, transported, and metabolized are rarely reported. Thus, it is so far impossible to give a single mechanism that explains all of the physiological functions of anthocyanins including Cy3Gal.

For Cy3Gal itself, there is discrepancy in the Cy3Gal bioactivity results between related studies in vitro and those in vivo, because of the complexity of metabolic routes [17,147,148,149,152,153,154]. Hence, when discussing the in vitro and in vivo antioxidant activities of dietary anthocyanins in relation to human health, it is necessary to take the abovementioned bioavailability, transformation during digestion, and metabolism into consideration [155].

#### 5.2.1. Acting Solely

How Cy3Gal purified from blueberries affects learning and memory of aged mice was evaluated using the Morris water maze and passive avoidance test in the study of Yang et al. [132]. Aged mice that received intragastric administration of Cy3Gal showed better cognitive performance. Moreover, through anatomic analysis, this was probably to some extent attributed to the increased SOD activity in the plasma and brain tissue, decreased levels of lipofuscin in brain tissue, and decreased malondialdehyde (MDA) in the plasma and brain tissue of the tested mice. Furthermore, the metabolites of plasma and brain tissue of aged mice receiving Cy3Gal in feed was investigated by the ^1^H-NMR technique and multivariate analysis in these authors’ later study [122]. The results showed that dietary supplementation with Cy3Gal elevated the level of choline in plasma of mice, but reduced the levels of lactate and phosphocholine; and it increased the level of taurine in the brain of mice. Since these metabolites act as markers of a series of physiological changes parallel with aging (e.g., oxidative stress increase, acetylcholine release in nervous system), it is believed such results highlighted the ability of Cy3Gal to improve cardiovascular diseases, cognitive impairment, and neurodegenerative diseases [122]. In the later study by Lee et al., oral administration of purified Cy3Gal could partly reverse scopolamine-induced spatial memory impairment on mice evaluated by the Morris water maze test and passive avoidance test [133]. These cognitive protective effects of Cy3Gal were deemed to correlate with the inhibited acetylcholinesterase activity, enhanced brain-derived neurotrophic factor (BDNF) expression, and cAMP-response element binding protein (CREB) phosphorylation in the hippocampi of the aging mice [133].

Correlatively, in the recent work by Skemiene et al. on rat brains, experiments through monitoring the caspase 3 activity and LDH release, as well as the expansion of infarct area in cerebral cortex and cerebellum in ischemia-damaged rat brains, indicated the necrosis and apoptosis-inhibiting ability of Cy3Gal [18]. These protective effects of Cy3Gal upon rat brains were, however, attributed to the reductive, rather than antioxidant capacity of Cy3Gal [18]. Moreover, in the recent studies by Wen et al., further examination of the coronal hippocampal sections of rats using Nissl staining, Cy3Gal was found to have a strong ability to ameliorate learning and memory impairment among amyloid-β-induced neurotoxicity rats [19]. Such results highlighted the potential of Cy3Gal to effectively deal with Alzheimer’s disease.

Other anthocyanins were also proven to exhibit protective effects against cognitive impairment by in vivo studies [18,156,157]. However, there are very few studies to directly compare the positive effects between Cy3Gal and other anthocyanins. In the study of Skemiene et al., a higher brain protective effect of Cy3Gal was found when compared to Cy3Glu and cyanidin 3-rutinoside (Cy3Rut). It was observed that Cy3Gal required 10 μM to achieve its protective effects against ischemia-induced caspase activation and necrotic cell death in rats, while Cy3Glu required 20 μM and Cy3Rut displayed no effect in neither concentration [18].

#### 5.2.2. Acting Synergistically

The in vivo assessment of the health effects of Cy3Gal enriched plants is multifaceted between individual studies, which mainly focused on their antioxidant, cardiovascular protective, anti-diabetes, anti-inflammatory, and anticancer effects.

In the animal study of Maslov et al., administration of chokeberry extract was capable of reducing serum glucose levels in streptozotocin (STZ)-diabetic and normal rats [134]. In a human study, Pilaczynska-Szczesniak et al. discovered that chokeberry juice consumption improved the serum antioxidant status of rowers [102]. Moreover, in the study of Broncel et al., patients with metabolic syndrome (MS) who regularly received chokeberry extracts exhibited significant decreases in LDL cholesterol and triglycerides levels in their serum, while the level of high-density lipoprotein (HDL) cholesterol did not change significantly [103]. Beyond these, increases in levels of SOD activity, glutathione peroxidase (GSH-Px), and decreases in the levels of lipid peroxidation in erythrocytes were observed in these patients. However, on the other hand, the level of CAT activity was unexpectedly inhibited by the treatment [103]. Furthermore, according to the studies of Broncel et al., Hellström et al. and Sikora et al., chokeberry polyphenols are capable of inhibiting the angiotensin I-converting enzyme (ACE) activity both in vitro and in vivo, and lowering the blood pressure of both spontaneously hypertensive rats and patients with MS [103,119,124]. In addition to the further-observed decreases in the serum level of endothelin-1 in patients in the study of Broncel et al., and the high correlations between the ACE activity and blood pressure in the study of Sikora et al., these suggests a favorable hypotensive action of chokeberry polyphenols [103,119]. In the work of Poreba et al., men with mild hypercholesterolemia and received chronic treatment with chokeberry juice showed significant decreases in their serum total cholesterol, LDL cholesterol, and triglycerides levels [120]. A statistically significant increase in serum NO concentration and in the flow-mediated dilatation (FMD) was also observed. These indicated that chronic intake with chokeberry juice improves vascular function and reduces cardiovascular remodeling by increasing NO release from endothelial cells. The underlying mechanism of this hypolipidemic effects of chokeberry polyphenols were investigated in the later in vitro study by Kim et al. at the protein and gene levels with caco-2 cells [158]. It was suggested that such effects were owing to the elevated apical efflux of LDL-derived cholesterol and decreased chylomicron formation in the intestine.

By determining the number and area of gastric mucosal lesions and the levels of malondialdehyde (MDA) in the rat plasma and gastric mucosa, as well as the levels of reduced glutathione (GSH) and oxidized glutathione (GSSG) in gastric mucosa of pretreated rats, the work of Valcheva-Kuzmanova et al. revealed that oral intake of chokeberry juice could diminish the indomethacin-induced gastric mucosal damage in the studied rats, and interfere with the induced oxidative stress development (evidenced by the level of MDA) in rat plasma and gastric mucosa [136]. In their further study, chokeberry juice also exhibited its potential in preventing diabetes mellitus and diabetes-associated complications, as observed that chokeberry juice applied by gavage reduced the plasma glucose and plasma triglycerides in streptozotocin-induced diabetic rats [135]. Similarly, in the studies of Qin and Anderson, with the supplement of chokeberry extract added to the drinking-water, decreases in epididymal fat, blood glucose, triacylglycerol (TAG), cholesterol, and LDL-cholesterol, as well as plasma TNF-α and IL-6 were observed in rats fed a fructose-rich diet [127]. An increase in rat plasma adiponectin was also significant. Additionally, the influences of chokeberry extracts on relevant protein and gene expression in the epididymal adipose tissues of rats were also investigated by the same group. To be specific, in the presence of Cy3Gal, changes were witnessed in the expression of genes related to insulin signaling (increases in *Irs1*, *Irs2*, *Pi3k*, *Glut1*, *Glut4,* and *Gys1* and decrease in *Gsk3b*, at mRNA levels), genes involved in adipogenesis (increases in *AdipoQ* at mRNA and protein levels, and *Pparg* at mRNA levels, and decreases in *Fabp4*, *Fas,* and *Lpl* at mRNA levels), and genes of inflammatory cytokines (increase in *ZFP36* at mRNA and protein levels, decreases in *Il1b*, *Il6,* and *Tnfa* at mRNA levels), respectively [127]. Such results at a molecular level are in agreement with the human study of Naruszewicz et al., in which chronic chokeberry extract consumption reduced the severity of plasma inflammation, increased the anti-inflammation factor, and plasma adiponectin levels [121]. Takahashi et al. studied the effects of intake of anthocyanin-enriched chokeberry extract on KBrO_3_-induced renal damage in rats [116]. They found that the blood urea nitrogen (BUN), serum creatinine, aspartate aminotransferase (AST), and alanine aminotransferase (ALT) activities of the studied rats supplied with chokeberry extracts were markedly lower than those of the control rats. Meanwhile, chokeberry extracts reversed decreases in oxygen radical absorbance capacity (ORAC) levels of serum and kidneys, and KBrO_3_-induced increases in thiobarbituric acid reactive substance (cholesterol) level of kidneys. These results indicate that the chokeberry extract was capable of suppressing KBrO_3_-induced renal and hepatic damage to rats in vivo [116].

The effects of chokeberry polyphenol consumption on healthy human body were also evaluated in the study of Kardum et al. [104]. After regular dietary intervention with chokeberry juice, subjects (female volunteers that were apparently healthy) exhibited important decreases in TBARS, pro-oxidant antioxidant balance (PAB), and increases in paraoxonase-1 (HDL-associated esterase that help protect against atherosclerosis) activity in the venous blood of the subjects. However, the blood total antioxidative capacity (TAC) value was significantly lower than the baseline [104].

The in vivo anticancer capacity of chokeberry extract was reported by Lala et al. [131]. It was observed that consumption of chokeberry extract inhibited the formation of the azoxymethane-induced aberrant crypt foci, a tentative marker of dysplasia and malignant transformation, and decreased the colonic epithelial cell proliferation rate as well as the fecal bile acid concentration in rats [131]. Nonetheless, the in vivo study about the anticancer effects of consumption of chokeberry or chokeberry products in humans is rarely reported.

## 6. Potential Applications

Considering the aforementioned physiological functions of Cy3Gal, and its other bioactivities that are universal among anthocyanins, Cy3Gal or plant materials containing high amount of Cy3Gal exhibit high potential to be ideal dietary supplement. However, few studies explored the potential applications of isolated or purified Cy3Gal except for those performed to assess its bio-functionalities related to health issues. On the contrary, most of the research is conducted using Cy3Gal containing plants or their products. The work of Rupasinghe et al. investigated the feasibility of using red-fleshed apple to make functional beverages [12]. It was reported that the concentrations of Cy3Gal in the juice made from the selected red-fleshed apple reached 39 mg/L, and the juice also exhibited relatively high antioxidant capacity in the in vitro Ferric ion reducing antioxidant power (FRAP) and ORAC assays. In addition to the in vivo bioactivities discussed previously, this indicates that Cy3Gal may be a good nutrition enhancer for beverage making, or therapeutic supplement. On the other hand, the correlations between anthocyanins and colors were found in the previous studies [1]. According to the studies of Han et al., Zatylny et al. and Li et al., Cy3Gal is most likely to correlate with the dark red or dark purple of the studied plant materials [87,97,159]. These suggests that Cy3Gal can be also used as a pigment for food coloring, though its stability under the storage conditions of many food products is still up for improvement. Since acylation of the anthocyanins enhances their stability by means of intramolecular and/or intermolecular co-pigmentation and self-association reactions, enzymes that promote the acylation of Cy3Gal may provide the desirable stability of Cy3Gal for food coloring applications [160].

## 7. Conclusions

Anthocyanins originate from plants, and are proven to have ideal health effects on animals and humans; hence, they receive great attention from the public and the scientific community. Here, we reviewed previous studies on one of the widespread anthocyanins, Cy3Gal, as it is enriched in many foods from plant sources, especially fruits, such as apples and berries of the human diet. Cy3Gal is biosynthesized from cyanidin and UDP-galactose, of which the former adopts a general synthesis pathway of anthocyanins with phenylalanine as the starting compound, followed by a series of reactions, including deamination, hydroxylation, dehydration, condensation, cyclization, methylation, and glycosylation. Moreover, the latter is obtained from the intermediate chemicals in the biosynthesis of polysaccharides. Thus, enzymes participating in the pathway and their corresponding genes are the key factors regulating the biosynthesis of Cy3Gal, where UCGalT plays an essential role during the regulation. The chemical stability of Cy3Gal is similar with other anthocyanins: it is to varying degrees affected by temperature, acidity, humidity, light exposure, and gas composition, as well as the presence of mental ions and other exogenous additives. The extraction methods for qualitative and quantitative analysis of Cy3Gal are somehow conventional. Organic solvents are often used as the transfer media based on their polarity and that of the anthocyanins. The content of Cy3Gal in different plants varies with their species, even though the most ideal sources for mass-extraction of Cy3Gal seem to be chokeberries, bilberries, and crowberries. As many other anthocyanins, Cy3Gal also possesses remarkable antioxidant capability, from which other physiological functions of Cy3Gal were derived. These include its cardiovascular protective and nervous protective abilities, in terms of the studies performed with either purified Cy3Gal or products from its enriched plants. Moreover, the anti-inflammatory, anticancer, antidiabetic, and anti-toxicity of poisonous substance were also confirmed in the plant extracts, but whether these are to some extent due to the presence of Cy3Gal in the mixture is still in need of further evaluation. All of the mentioned desirable chemical properties of Cy3Gal provide the possibility of exploring its potential application in both the food and medicine industries, mainly in nutrition supplements and coloring.

As mentioned above, the physiological functions of purified Cy3Gal still needs further investigation. A greater depth of understanding of Cy3Gal may lead to an increase in the demand for mass-production of this compound. However, traditional extraction methods using plants as the extraction source are inefficient. Moreover, the low time- and energy-consuming chemical synthesis in vitro of anthocyanins is still impossible; hence, their biosynthesis is worth developing. Heterologous expression of certain genes was already applied in previous studies to enhance the accumulation of anthocyanins in the original plants. Alternatively, heterologous biosynthesis of either anthocyanin mixture or individual anthocyanins using metabolically engineered microbes was also previously highlighted. It is worth it to mention that these artificially modified biosynthesis technologies were studied largely with respect of the production of glucosylated anthocyanidins (Cy3Glu in particular). Assessment of the feasibility of these methods to massively produce Cy3Gal in vivo is still scarce. On the other hand, the high in vitro activity of the recombinant UCGalT from *Escherichia coli* BL21 cells also provides new potential strategies to directly generate Cy3Gal. Such strategies could combine both in vitro and in vivo methods with the supplementation of cyanidin and UDP-galactose, though further evaluation for the optimal application conditions to achieve high efficiency is still necessary.

Despite that, the mechanism of the physiological functions of Cy3Gal is attributable to its antioxidant effect; it is still in need of elaboration on the other possible underlying mechanisms against Alzheimer’s. Mechanisms explained via the combination of the structure and functions of the compound, as well as the metabolism of the organism, should be emphasized in future studies, as well as developing new technologies that can stabilize Cy3Gal in food or beverages, exploring its application in functional products.

## Figures and Tables

**Table 1:** Contents of Cy3Gal from different natural sources ^a^.

Direct Sources	Botanical Name	Content of Cy3Gal (mg/100g Material Weight)	Notes	Reference
Achene	*Coriaria myrtifolia* L.	607.3 ^c^	-	[74]
Apple juice	*Malus pumila*	12.5 ^d^	-	[12]
	*Malus marjorensis*	1.25 ^d^		
Apple red flesh	*-*	6.765 ^b^	-	[11]
	*Malus domestica*	0.45–47.63 ^b^	-	[75]
	*Malus* spp.	31.86–1246.36 ^c^	The whole fruit was used in the study	[76]
Apple white flesh	*Malus domestica*	0.00 ^b^	-	[75]
	*Malus micromalus*	0.00 ^c^	The whole fruit was used in the study	[76]
Apple peel	*Malus domestica*	4.9321–85.6 ^c^	-	[73,77]
		0–20.82 ^b^	-	[11,78]
Bilberry	*Vaccinium myrtillus*	370 ^b^	-	[79]
Black soybean seed coat	*Glycine max* L. Merr.	6.0 ± 3.6 ^b^	The data are counted as the mean of 60 varieties	[80]
Blueberry	*Vaccinium* spp.	28 ^b^	-	[79]
	*Vaccinium corymbosum* L.	6.24 ± 0.81 ^b^	-	[81]
Chokeberry	*Aronia melanocarpa* Michx.	168.0–636.0 ^b^	-	[82,83,84,85]
	*Aronia prunifolia*	497 ± 10 ^b^	-	[85]
Chokeberry juice	*-*	108.9–326.1 ^d^	-	[67]
Cornelian cherry fruit	*Cornus mas* L.	4.63–130.93 ^b^	-	[86]
Crabapple flower petal	*Malus hupehensis*	131.37–1430.75 ^c^	-	[87]
Cranberry	*Vaccinium macrocarpon*	5.7–123.4 ^c^	Materials were dried using different methods	[64]
	*Vaccinium oxycoccos*	386 ^b^	-	[79]
Cranberry juice	*Vaccinium macrocarpon* Ait.	64.20 ^d^	-	[15]
Crowberry	*Empetrum nigrum*	804 ^b^	-	[79]
Deerberry	*Vaccinium stamineum* L.	74.42–98.75 ^b^	-	[88]
Hawthorn fruit	*Crataegus pinnatifida* var. major	15 ± 1.10 ^c^	The data are the mean from 10 cultivars	[13]
	*Crataegus brettschneideri*	37 ± 3.36 ^c^	The data are the mean from 8 cultivars	
	*Crataegus pinnatifida*	20 ± 2.89 ^c^	The data are the mean from 3 cultivars	
	*Crataegus scabrifolia*	0 ^c^	-	
				
Lingonberry	*Vaccinium vitis-idaea* L.	18.7–35.5 ^b^	-	[89]
		575 ± 20 ^c^	-	[90]
Kenyan tea	*Camellia sinensis* L.	0.951–15.544 ^d^	-	[91]
Pear peel	*Pyrus communis*	23.7 ± 3.2 ^b^	-	[92]
Pistachio nuts skin	*Pistacia Vera* L.	69.6 ± 4.5 ^b^	-	[93]
Poinsettia bract	*Euphorbia pulcherrima* Willd.	0.724–108.168 ^b^	Only data of bracts in fully pigmented stage are presented	[94]
Purple wheat	*Triticum aestivum*	0.098 ± 0.015 ^b^	-	[95]
Saskatoon berry	*Amelanchier alnifolia* Nutt.	20.5–155.0 ^b^	-	[96,97]
	*Amelanchier arborea*	390 ^b^	-	[98]
	*Amelanchier canadensis*	165 ^b^		
Strawberry	*Fragaria × ananassa*	0.214 ± 0.0014 ^b^	-	[99]
	*Arbutus unedo* L.	2.84 ± 0.540 ^b^	-	[100]
Theaceae flower petal	*Camellia reticulata*	19.5 ^c^	The fresh petals were treated with boiling water for 4–6 s and were then dried at room temperature	[101]
	*Camellia pitardii* var. yunnanica	33.1 ^c^		

^a^ Information from previous studies is not exhaustive because of the context limit. Number after each “±” represents standard error (some are converted from standard deviation, but not all variation is given in the original). ^b^ Material weight was counted on a fresh weight basis. ^c^ Material weight was counted on a dry weight basis. ^d^ For liquid materials, the data units are “mg/L”. “-” represents “none”.

## Data Availability

No new data were created or analyzed in this study. Data sharing is not applicable to this article.

## Supplementary Materials

The following are available online at https://www.mdpi.com/1422-0067/22/5/2261/s1, Figure S1: Alignment of deduced amino acid sequences of galactosyltransferases from *Actinidia chinensis* (Accession No. GU079683), *Aralia cordata* (Accession No. AB103471), *Apium graveolens* (Accession No. MG725374), *Daucus carota* (Accession No. KP319022), *Malus domestica* (Accession No. NC041789), *Petunia hybrida* (Accession No. AF165148) and *Vigna mungo* (Accession No. AB009370), respectively.
