# Supplementary material for: Cyanidin 3-O-galactoside: A Natural Compound with Multiple Health Benefits
Source: Int J Mol Sci. 2021 Feb 24;22(5):2261. doi: 10.3390/ijms22052261 (PMC7956414; doi:10.3390/ijms22052261)
Supplement: Supplementary file 1 [file ijms-22-02261-s001.pdf]

```
A.chinensis     ............................................................
A.cordata       ............................................................
A.graveolens    ............................................................
D.carota        ............................................................
M.domestica     ATTAGCCTTGTAAGCTGTAATGGCAGCGCCGCTGCCCATCGAAATCGAACCATCATCAAC
P.hybrida       ............................................................
V.mungo         ............................................................

A.chinensis     ............................................................
A.cordata       ............................................................
A.graveolens    ............................................................
D.carota        ............................................................
M.domestica     TAATGGTCAACCCCATCTCGCCGACGCCTACAACCGTCACGTGGCTGTCGTAGCCTTCCC
P.hybrida       ............................................................
V.mungo         ............................................................

A.chinensis     ............................................................
A.cordata       ............................................................
A.graveolens    ............................................................
D.carota        ............................................................
M.domestica     TTTCACTAGCCATGCAAGCGCCTTGCTTGAGACCGTGCGCCGCCTAGCCACCGCCCTTCC
P.hybrida       ............................................................
V.mungo         ............................................................

A.chinensis     ............................................................
A.cordata       ........TCTTCACTAGTCACTAGACTACTATACAACTTGGATATATATATATGTATGT
A.graveolens    ............................................................
D.carota        ............................................................
M.domestica     AAACACTCTCTTCTCGTTCTTCAGCACTTCAAAATCCAACAGCTCTCTCTTTTCCAACAA
P.hybrida       ............................................................
V.mungo         .........................................................AAT

A.chinensis     ............................................................
A.cordata       ATTATATCCCTTATTTATTCATAAGTTAATTCAATCTGTGCTAGTTACCTATCTCTCTTC
A.graveolens    ............................................................
D.carota        ............................................................
M.domestica     CAGCATTGATAACATGCCGCGTAACATAAGGGTGTACGATGTGGCTGACGGGGTGCCGGA
P.hybrida       ............................................................
V.mungo         TCGGCACGAGTATTGGTGGAATCAAGAATGTATATATTTAAGTGTGAACAGAGCTACTAA

                                                     1       10        20
A.chinensis     .....................................ATGCTAATTACCAAATCCTCATC
A.cordata       TGGAATTCGAGAATTAATCAGCAGGTGGAATAATTAATTATTAATGGGGAGTTCAG...C
A.graveolens    ...........................................ATGGGTAGTACAAATAT
D.carota        ...........................................ATGGGGAGTACAAATCT
M.domestica     GGGGTACGTTTTCGTGGGCAAGCCGCAGGAGGACATAGAGCTCTTCATGAATGCCGCACC
P.hybrida       ...........................................TTGTCCAAAATGT...C
V.mungo         ACTCCATTGCTCTCATTTTTCTCAGATCAAGGCACAGCAAATCATGGGAAATTCAGAAGA

                     30        40        50        60                70
A.chinensis     TGAATGCCACGTGGCCGTGCTCGCATTCCCGTTTTCCACGCAC......CCCGGCCCCCT
A.cordata       GGAGCCACATGTGGGGGTCCTTGCCTTCCCATTTGCTACACAC......GCCGGTCTCCT
A.graveolens    GGAACCCCATGTAGCCGTTCTTGTTTTTCCGTTTGCTACCCAC......GCTGGTCTCCT
D.carota        GGAACCCCATGTCGCGGTTCTTGTTTTTCCATTTGCCACCCAC......GCTGGTCTGCT
M.domestica     GGAA.AACATCCGGAGGAGCTTAGACGCTTCCGTGGCGGACATCGGGAAGCAGATCAGCT
P.hybrida       CAATTACCATGTTGCTGTTCTAGCATTTCCTTTTGCAACACAT......GCTGGCCTTTT
V.mungo         GAAGAAACATGTTGCAGTGTTTTCTTTCCCCTTTGGAAGCCAC......CCTACTCCTCT
```

```
                      80        90        100       110       120
A.chinensis   CCTCAACC..............TTGTCCAACGGCTGGCGGT.CGAGGCCCTGACGTGA
A.cordata     CCTGGGCC..............TAGTCCGTAGGCTAGCCGC.CGCTGCCCTAACGTCA
A.graveolens  CTTTGGCC..............TCGTACAACGGTTGGCCAG.GGCTGCTCCAAATGTGA
D.carota      CTTTGGCC..............TCGTGCAGCGTCTGGCTAA.GGCTGCACCGAATGTCA
M.domestica   GCTTGATCACCGACGCCTTCCTTGGTTTGGAGTCCACTTGGCTGACGAGTTGGGAGTGC
P.hybrida     ACTTGGAC..............TTGTACAAAGGCTAGCAAA.TGCATTACCTAACGTGA
V.mungo       GTTAAACC..............TGGTGCTGAAGCTGACCAA.TGCTGCTCCCAACTTGC

                     130       140       150       160       170
A.chinensis   TCTTC.TCTTTCATTAGCACGGCCAAGTCGAACGAGTCCTT...GTTCTCAGTGCCAAA.
A.cordata     ATTTC.TCTTTCTATAGCACCGCTGCATCCAACCGTTCATT...ATTTTCATATCCAAAT
A.graveolens  AGTTC.ACATTTTTAAACACAGCCAAGTCTAACCACACATT...GTTTTCAGATTTATCA
D.carota      AGTTC.ACATTTTTTAACACGGCCAAGTCGAACCATTCGTC...GTTTTCAAATTCATCA
M.domestica   CTTGGGTCACTTTCTGGATCTCCGGACTCAAATCCCTCTCCG.TTCATGTGCATACTGAT
P.hybrida     CATTT.ACATTCTTTAACACGTCCAAATCAAATTCTTCATT...ATTCACTACTCCTCA.
V.mungo       AATTC.TCATTCATAGGCACCGAACACTCCAACAAATCTCTTCTGATCTCAAAGCCCCA.

                     180       190       200       210       220       230
A.chinensis   .....CCCTGAGAACATAAAGCCTTACCCCGTTTGGGACGGCGTTCCAGAGGGATACGTG
A.cordata     TCTCCTTATTCTAATGTAATACCCTACGATGTCTCCGATGGCGTACCAGAGGGCTACGTG
A.graveolens  ACTGTTGCTAGTAATATAACTCCATTTGATGTTTATGATGGCGTGGAGGCCGGATATGTG
D.carota      AGCATTGCTAGCAATGTTATACCTTATGATGTCTATGATGGCGTGGAGGAGGGGTATGTG
M.domestica   CTCATCCGCGACACTATTGGAACTCAAGGTGCGTACGTTATACTTACACTTACTTATCCT
P.hybrida     .....TGACAACAACATTAAACCGTTTAATATCTCCGATGGCGTCCCGGAGGGTTACGTG
V.mungo       ..CATCCCAGACACCATCAAGTTCTACAGTATAAGCGATGGAGTCCCAGAGGGTCA..TG

                       240       250       260       270       280
A.chinensis   TTCGCGG..GGAAGCCCC.AGGAAGACATTAACTTGTTCCTTAAGGTGGCGGCAAAGGGC
A.cordata     TTTTCCG..GGAAGCCAC.AAGAGGATATTAACTTGTTCTTGACCGTTGCCTCCGATGAA
A.graveolens  TTCTCTG..GGAAACCTC.AAGAGGATATTAGCTTGTTCTTGGCTGTGGCTGCGGATGAG
D.carota      TTCTCGG..GGAAGCCAC.AAGAGGATATCAACTTGTTCCTGGCTGTGGCAGCGGATGAG
M.domestica   GTCTTTAA.AGTTGAAGCATTGAGAATCCTTGTTCGTTTGGTAGTACTTATCGAGAAAGC
P.hybrida     GTCGGAAAAGGAGGTATTGAAGCGCTTATAGGGTTGTTCTTTAAGTCTGCTAAAGAGAAT
V.mungo       TTCCAGGT.GGCCATCCAGTCGAAAGAGTCAACCTTTTTCTTCAGGCTTCTCCTCAGAAC

                     290       300       310       320       330       340
A.chinensis   .......TTTAAGTTGGCGATGCAGGCGGTTGAAGTAGAGACAGGCGGAGGATAGGCTG
A.cordata     .......TTTAAGAGAGGCTTGGAAAAAGCCGCGGTGGACTCCGGTAGGAAGATCACCTG
A.graveolens  .......TTCCGGCGAGGTGTGGAGAAGGCAGCTGCGGATTCTGGTAGCAAGATCACTTG
D.carota      .......TTTCGACGAGGCTTGGAGAAGGCAGTTGTGGATTCTGGTAGGCAGATCAGTTG
M.domestica   ATTTTTCTTTCAAGTGTTTATTGGAAAAGCACTTTCCAGTCACAAATAGGTGCTTCACTT
P.hybrida     .......ATTCAAAATGCTATGGCAGCTGCTGTGGAGGAATCGGGAAAGAAGATTACTTG
V.mungo       .......TTGCAAAAGGGAATAGACATGGCAGTGGCTCACACAAAAGAGAGAGTTACTTG

                     350       360       370       380       390       400
A.chinensis   GGTGATGGCGGACGCCTTTTGTGGTTTTCTAGCGACATGGCGGAGGAGAGGGGCATCCC
A.cordata     TTTGGTGGCGGACGCTTTTTTATGGTTTTCCGGCGATTTGGCCGAACAAATTCGTGTGCC
A.graveolens  TTTGGTTGCGGACGCGTTTTTGTGGTTTTCTTGTGATTTGGCACAAGAGATTGGTGTGCC
D.carota      TTTAGTTGCAGATGCATTTTTGTGGTTTTCTTGTGATTTGGCACAAGAGATTGGTGTGCC
M.domestica   TTCCTTTTTAAATATTTCTCT.....CATTTATCAACAT.ACATAATGGTTATTTATTAC
P.hybrida     TGTTATGGCAGATGCATTTATGTGGTTTTCTGGTGAGATTGCTGAGGAATTGAGCGTTGG
V.mungo       TGTCATCTCTGATGCTTTTGTCGCACCTTCTCTCACTGTGGCTCAGCGCTTGAACGTTCC

                     410       420       430       440       450       460
A.chinensis   GTGGGTGCCAATTTGGATGTCAGGGGCGTGCTCCTTGTCGGTACATTTGTACACTGACCT
A.cordata     ATGGGTCCCACTTTGGACTTCCGGAGCTTGCTCGCTTTCGATCCACGTTTACACCGATCT
A.graveolens  ATGGGTGCCACTTTGGACCTCCGGGGCTTGCTCTCTTTCAACTCACATTTATACTGATCT
D.carota      ATGGGTGCCACTTTGGACCTCCGGGGCTTGCTCTCTTTCAACTCACATTTATACTGATCT
M.domestica   TTGGATTTCAAGTTTAAGTATCGAATGATTTCAATTTTAGGTTTAGTATCA..AATTT
P.hybrida     TTGGATCCCTTTATGGACTTCTGCTGCTGGATCACTCTCTGTTCATGTTTACACTGATCT
V.mungo       ATGGGTTCCGGTTTGGCCTCCTTTGTCATGCTCACTCTCTGCGCATTTTTACACTGAATT
```

```
                    470       480       490       500       510       520
A.chinensis   TATTAGGGAAACAGTAGGATTTAGTGGAATTTCTGGACGCCAAAACGAACTCTTGAAATT
A.cordata     TATCAGGCAAACTGTTGGACTTGGTGGTATTGAAGGACGTATGGATGAGATTCTGACGTT
A.graveolens  TATTCGCCAAACTGTTGGACTAGAAGGTGTAGAAGGACGTATGAATGAAAAGCTAAACTT
D.carota      TATTAGGCAAACTGTTGGATTTGATGGAATTGAAGGACGTATGGATGAGAAGCTGAACTT
M.domestica   TGTGCATTCAGGCATTACAGGTCGTGAAAACGACCTCATCGTCGACAAAAATGTTAA...
P.hybrida     CATTAGAGAAAATGTTGAGGCTCAAGGAATTGCTGGACGTGAAGATGAAATTCTAACATT
V.mungo       GATACGCCAAAC.GTGTAACTCTGCAGCA............GGAGACACCCCTTGGATTT
```

```
                    530       540       550       560       570
A.chinensis   CATCCCAGGATTTTCCGAGCTGCGCCTCGGTGACTTGCCCAGTGGAGTCTTGC......T
A.cordata     CATTCCCGGATTTTCTGAATTACGGCTCGGTGACTTACCCGGTGGAGTCCTTT......T
A.graveolens  CATTCCTGGATTTTTTGAACTAAGATTAGGTGACTTGCCTGGTGGAGTGGTGT......T
D.carota      CATTCCTGGATATTCTAATTTAAGGCTAGGTGACTTGCCTGGTGGAGTGGTGT......T
M.domestica   CATCCAAGGTCTCTCCAATGTACGAATCAAAGACTTAGCGGAAGGAGTCATTT......T
P.hybrida     CATTCCAGGATTTGCCGAATTACGGCTAGGTAGTTTGCCTAGTGGAGTTGTCT......C
V.mungo       TGTTCCTGGATTGTCTAAGATGCGAGTTGAAGACTTGCCAGAAGATGTGATCCAAGGTGC
```

```
                580       590       600       610       620       630
A.chinensis   TGGAAACTTGAAATCCCCATTCTCAATCATGCTACACAAAATCGGACAAGCTCTTCCAAA
A.cordata     CGGTAACTTAGAATCACCCTTCTCAATAATGCTACATAAAATGGGACAAACTCTACCCCG
A.graveolens  TGGGAACTTAGAGTCTCCTTTCTCTGTAATGCTACATAAAATGGGGCAAACATTACCCGG
D.carota      TGGAAATCTAGAGTCTCCTTTCTCTGTAATGCTACATAAAATGGGACAAGTATTACCCCG
M.domestica   CGGAAACTTGGACTCGGTAATTTCCGGCATGCTACTTCAGATGGGACGGCTCCTCCCCCG
P.hybrida     TGGAGACTTGGAATCACCATTTTCCGTAATGCTTCACAAAATGGGCAAAACAATAGGAAA
V.mungo       CGGTGAAGAGGAGACATTGTTTTCGAAGACACTAGCATCGTTGGGAAGTGTGTTACCTCA
```

```
                640       650       660       670          680       690
A.chinensis   AGCAACCACTGTTCTAATTAACTCCTTCGAAGAACTTGACCC...TGAACTCAATAAAGT
A.cordata     GGCAGCTGCCGTTCCCATTAACTCCTTCGAAGAGCTTGATCC...TGATCTCATGAAAGA
A.graveolens  GGCATGTGTTCTGGCAATGAACTCATTCGAAGAACTAGACCC...TGACCTCATGAAAGA
D.carota      AGCAGATGTTCTGGTGATGAACTCATTCGAGGAACTAGACCC...TGACCTTATGAAAGA
M.domestica   TGCCACCGCAGTTTTCATGAACGGCTTCGAAGAATTGGAACT...CCCCATACCAAACGA
P.hybrida     AGCCACTGCCTTGCCAGTAAACTCCTTTGAAGAACTAGACCC...TCCAATTGTTGAAGA
V.mungo       AGCTGAGGCAGTGGTTGTGAATTTTTTTGAGGAACTAGATCCACCCTTGTTGGTTAACGA
```

```
                   700       710       720       730       740
A.chinensis   TCTCAACTCCAACTTTGGGAAGTTCCTCAACATCGGCCCGTCCAACCTAACATCACC...
A.cordata     TATAAAATCAAAGTTCAAAAAAATCCTCAACGTCGGTCCCTTCAACCTAACATCGCC...
A.graveolens  TCTTAGTTCAAAGTTCAACAAGATTCTTAATGTAGGACCTTTTAACCTTACATCACC...
D.carota      TCTGAGTTCAAAGTTCAAAAAGATTCTCAATGTAGGCCCTTTTAACCTTACATCACC...
M.domestica   CCTAAAGTCCAAAGTCAACAAACTCCTCAACGTAGGACCTTCCAACGTAGCATCCCCGCT
P.hybrida     TCTCAAGTCCAAATTCAATAACTTCCTCAATGTTGGTCCCTTTAACTTAACTACCCC...
V.mungo       CATGAAGTCAAAGTTTAAGTATTATCTTTATGTTGGTTTTCTCACTCTATCACTGCCTCT
```

```
            750       760             770       780       790       800
A.chinensis   ACATCCATTATC...AAA...TTCCGACGAGTATGGATGCATCCCATGGCTAGCCAAGCA
A.cordata     ACCACCATCATC...AAA...CTCCGACGAACACGGCTGCATACCGTGGTTAGACAACCA
A.graveolens  ACCAGCATTACA...ATA...TTCAGATGAATATGGCTGCATACCGTGGCTAGACAAGCA
D.carota      ACCAGCATCACA...ATA...CTCAGATGAATATGGCTGCATACCATGGCTAGACAAGCG
M.domestica   GCCACCGCTGCC..........GCCATCAGA..TGCTTGCTTGTCATGGCTAGACAAGCA
P.hybrida     ACCACCCTCAGC...AAACATTACAGATGAATATGGGTGCATTGCATGGCTAGACAAACA
V.mungo       GCCACCCTTGCCTCCATCAGACACAGACGAAACTGGGTGCTTGTCATGGTTGGACAAGCA
```

```
                  810          820       830       840       850       860
A.chinensis   GAGATCCGCTTCC...GTGGCATATATTGGCTTTGGATCAGTAGCAAAACCGAAACCTGA
A.cordata     AAACCCTAAATCG...GTAGCGTATATTGCCTTCGGAACCGTAGCAACGCCGCCGCCGAA
A.graveolens  AAACCCCAGTTCT...GTGGCCTACATAGGCTTTGGAACTGTAGCGATGCTTCCTCCAAA
D.carota      TAATCCTAAGTCT...GTGGCCTATATAGGCTTCGGGACTGTGGCCATGCTTCCTCCAAA
M.domestica   ACAGGCTCCATCCTCCGTCGTGTACATAAGCTTCGGGACAGTGGCGAGCCCAGCGGAGAA
P.hybrida     AGAACCAGGCTCA...GTGGCTTACATAGGATTTGGCACGGTGGCTACACCACCACCTAA
V.mungo       GAAAGGTGGATCA...GTGGTGTATGTTAGTTTTGGGACGGTGGTGACACCACCTCCACA
```

```
                     870       880       890       900       910       920
A.chinensis    TGAGGTCGTGGCAATAGCTGAAGCACTAGAAGCTAGCAGTACTCCATTTCTCTGGTCTCT
A.cordata      TGAGCTAGTTTCATTAGCTGAGGCGCTAGAAGAAAGTGGTACTCCATTTCTTTGGTCTTT
A.graveolens   TGAGATAGCTGAATTAGCTGGAGCCCTGGAGTCAAGTGGAACTCCATTTATATGGTCAAT
D.carota       TGAGATAGTTGAATTGGCTGAAGCTCTGGAATCAAGTGGTACTCCATTTGTTTGGTCACT
M.domestica    GGAGCAGATGGCAATAGCGGAGGCCCTGGAAGCCACCGGAGCACCCTTCTTGTGGTCTAT
P.hybrida      TGAGCTAAAAGCTATGGCTGAAGCACTTGAAGAAAGTAAAACTCCTTTTCTTTGGTCACT
V.mungo        TGAGATTGTGGCAGTGGCGGAAGCGTTGGAAGCAAGTGGTTTTCCCTTTTTGTGGTCTCT

                     930       940       950       960       970
A.chinensis    TAGAGACACTTCGAAACAGTACTTACCAGAAGGATTCTTGAAGAGGAC.AAGCGAGCTA.
A.cordata      AAAGGACAATTTCAAAAATCATTTACCAAAAGGGTTTTTAGAAAGGAA.TAGTAAAAGT.
A.graveolens   GAAAGATCACTTGAAGCAACATTTGCCACCAGGATTTTTGGAAAGGAC.TACAGAAACT.
D.carota       GAAAGATCAGTCCAAGAAACATTTACCAGAAGGGTTTTTGGAAAGGAC.TAGAGAAAGC.
M.domestica    CAAGGACAGCTGCAAGACACCGTTGCTGAACGAGTTCTTGACAAAAACATTGTCAAAGCT
P.hybrida      TAAAGACCTTTTCAAATCATTTTTTCCAGAAGGTTTTTTGGAAAGAAC.TAGTGAGTAT.
V.mungo        GAAGGAGCATCTGAAGGGTGTTCTGCCAAATGGGTTTCTTGAGAGGAC.GAGTGAACGT.

                   980       990      1000      1010      1020      1030
A.chinensis    ....GGAAAAATTGTGCCATGGGCACCTCAGGTACAAGTTTTGGCCCATAGTTCAATTGG
A.cordata      ....GGGAAAATTGTGGCATGGGCACCCCAAATACAAGTTTTGTCACATGATGCCGTCGG
A.graveolens   ....GGAAAAATCGTGGCATGGGCACCACAAGTACAGGTTCTGTCTCATAGTGCTGTAGG
D.carota       ....GGAAAAATTGTGGCATGGGCACCCCAAGTGCAAGTTCTGTCACATAATGCCGTGGG
M.domestica    GAACGGGATGGTGGTGCCGTGGGCTCCACAGCCGCATGTACTGGCCCACGATTCGGTCGG
P.hybrida      ....GGTAAAATTGTGTCATGGGCACCACAAGTACAAGTTCTTAGTCATGGTTCAGTTGG
V.mungo        ....GGGAAAGTTGTGGGTTGGGCTCCTCAGACTCAGGTTTTGGGACATGGTTCTGTGGG

                  1040      1050      1060      1070      1080      1090
A.chinensis    GGTTTTCATAACTCATTGCGGGTGGAACTCGGTGTTAGAGACCATTGCGGGCGGTGTGCC
A.cordata      AGTTGTTATAACGCATGGCGGATGGAATTCGGTGGTGGAGAGCATTGCGGCCGGTGTGCC
A.graveolens   AATTGTTATAACGCATGGGGGATGGAACTCGGTGTTGGAGAGCATCGCAGCTGGCGTGCC
D.carota       AATTGTTATAACGCATGGGGGATGGAACTCGGTGCTGGAGAGCATTGCAGCTGGCGTGCC
M.domestica    AGCCTTCGTGTCGCATTGCGGCTGGAACTCGATAATGGAGACTATAGCAGGACGGGTGCC
P.hybrida      TGTTTTTATAAATCATTGTGGATGGAACTCTGTTTTGGAGAGTATAGCTGCTGGTGTGCC
V.mungo        AGTGTTTGTGACTCACTGCGGTTGTAACTCGGTGTTTGAGAGCATGTCGAATGGAGTGCC

                  1100      1110      1120      1130      1140      1150
A.chinensis    AATGATCGGTCGGCCATTCTTTGGGGACCACCCTATGAACACATGGATGGTGGAAAATGT
A.cordata      AGTGATATGTAGGCCGTTCTTCGGAGATCATCACATAAATACGTGGATGGTTGAAAATGT
A.graveolens   ACTGATCTGCAGACCGTTCTTTGGAGACCATTCAATAAACACATGGATGGTTGAAAATGT
D.carota       ACTCATCTGCAGACCCTTCTTCGGAGATCATGCAATAAACACTTGGATGGTTGAGAATGT
M.domestica    CATGATTTGTAGGCCATATTTTGCAGACCAGAGGCTTAATGCAAGGATGGTGGAGGAGGT
P.hybrida      TGTGATTTGTAGGCCATTTTTTGGAGATCATCAATTGAATGCTTGGATGGTTGAGAAAGT
V.mungo        TATGATATGCAGGCCTTTCTTTGGGGACCATGGGTTAACTGGGAGGATGGTTGAGGATGT

                  1160      1170      1180      1190      1200      1210
A.chinensis    GTGGAAAATTGGTGTGAGAGTTGAGGGTGGAGTGTTCACAAAGAGCAGTACAATGCGTGC
A.cordata      ATGGAAAATTGGTGTGAGAATTGAAGGTGGGGTTTTCACAAGAACTGGTACTATGAATGC
A.graveolens   ATGGAAAATTGGTGTGCAAATTAGTGGTGGTGTTTTTACCAAAGAAGGAACTGTACACGC
D.carota       ATGGAAAATTGGTGTGCGAATTAGTGGCGGTGTTTTCACCAAAAAGGGAACTGCAGATGC
M.domestica    GTTTGAGATCGGGGTAACCGTGGAGGATGGAGTTTTTACCAGGGAGGGGCTGGTAAAAAG
P.hybrida      ATGGAAAATTGGAGTGAAAATTGAAGGAGGAGTTTTTACTAAAGATGGAACAATGCTTGC
V.mungo        TTGGGAGATTGGTGTGAGAGTGGAAGGTGGGGTGTTCACCAAAGATGGGTTGCTGAAATC

                  1220         1230      1240         1250      1260
A.chinensis    ACTCGAACTGGT...TTTATCTCATGAAAAGGGAA...GAAATTGAAGGACCAAATTGG
A.cordata      GCTTGAACAAGTACTTTTGTCACAAGAAAAGGGAA...GAAATTGAAAGAGCAAATCAC
A.graveolens   CCTAGAACAAGT...TTTGTCGCAACAGAAAGGGA...AGAACTCAGAGAACAAATCAC
D.carota       CCTTGAACAAGT...TTTGTTGCGACAGAAAGGAA...AGAACTCAATCAACAAATTAC
M.domestica    CTTGGAAGTGGT...TTTGTCGCCTGAAAGTGGAG...GAAATTCAGAGACAATATAAA
P.hybrida      ACTTGACTTGGT...TTTATCAAAAGACAAAGGAATACAGAATTGAAACAACAAATTGG
V.mungo        CTTGAGGCTGAT...TCTGGTGGAGGAAGAGGGTAA...TCTCATGAAGAAAAATGCTGT
```

```
             1270      1280      1290      1300      1310      1320
A.chinensis  ACACTTAAGGGAGCTTGCTCTAAAGGCTGTTGGACCAAAAGGGAGCTCATCTCAAAACTT
A.cordata    AGTGTTTAAGGAGCTTGCTTTGAAGGCCGTTGGACCTAATGGCAGCTCTACTCAAAATTT
A.graveolens ATTGCTGAAGGATCTCGCATTCAAGGCTGTTGGACCTAATGGTAGCTCATCTCAAAATTT
D.carota     ATTACTCAAGGATCTTGCATTCAAGGCAGTTGGACCTAATGGTAGCTCATCTCTAAATTT
M.domestica  GAGGGTCAAACAACTGGCAGTAGAGGCGGTTGGACCACAAGGGAGCTCCACTCGGAACTT
P.hybrida    GATGTATAAAGAGCTTGCTCTAAATGCTGTTGGACCAAGTGGGAGCTCTGCTGAAAATTT
V.mungo      CAAAGTGAAAAAGACAGTGCTAGATGCAGCTGGAGCACAAGGTAAAGCAGCACAAGATTT

             1330      1340      1350      1360
A.chinensis  CAATAATTTGCTTGAGGTAATAACAGG.CCACAACCTTTGA...................
A.cordata    TAAACGCCTACTAGAAGTGATTACAAC.TTAATTTTTGTGGCATATGCACCTAGCTAGCT
A.graveolens TGAGGAGCTACTGAAAGAGATCACTAA.GTATGTACCAAATTAG................
D.carota     TACAGAGCTAGTAAAAGTGATTGCTGT.CTGA............................
M.domestica  CAAATCGCTGTTGGACATCGTATCAGGATCCAATTATCAAGTA..TAGTACGGGGACCAT
P.hybrida    CAAGAAATTGGTTGATATTATCACCTC.TTGCAATTAAAATTATTTACTTGAAGAGATTC
V.mungo      CAACACTTTGGTGGAACTGGTTTCTAGATCTTAATTTATGGCCCCTTTTGTCAGTGATAT

A.chinensis  ............................................................
A.cordata    TGTTGATGAATGAAGTTACCTTTTGTTATTAAAAAAAAAAAA..................
A.graveolens ............................................................
D.carota     ............................................................
M.domestica  AAATATGTAGCACT..............................................
P.hybrida    AATGATTTACTATCTGTTTGAAATAATAAATTCCACTTTAATTT................
V.mungo      TGATATATATCGCTGCCGTCAGAATGCAGTTTATGACCATGCAAGTGATTGTAATTTTTT

A.chinensis  ............................................................
A.cordata    ............................................................
A.graveolens ............................................................
D.carota     ............................................................
M.domestica  ............................................................
P.hybrida    ............................................................
V.mungo      TTACATGTTTTCTCTTGTTGAGGGTGAATAATAAGATCATTGTCTCATCTTTCATTGGTG

A.chinensis  .............................................
A.cordata    .............................................
A.graveolens .............................................
D.carota     .............................................
M.domestica  .............................................
P.hybrida    .............................................
V.mungo      GGAGTAGTTGACCAGAAATAAAAAGTGGCAAAAGTTGGAGACACC
```

Figure S1 Alignment of deduced amino acid sequences of galactosyltransferases from Actinidia chinensis (Accession No. GU079683), Aralia cordata (Accession No. AB103471), Apium graveolens (Accession No. MG725374), Daucus carota (Accession No. KP319022), Malus domestica (Accession No. NC041789), Petunia hybrida (Accession No. AF165148) and Vigna mungo (Accession No. AB009370), respectively.
